# Assembly and annotation of a non-model gastropod (*Nerita melanotragus)* transcriptome: a comparison of *De novo* assemblers

**DOI:** 10.1186/1756-0500-7-488

**Published:** 2014-08-01

**Authors:** Shorash Amin, Peter J Prentis, Edward K Gilding, Ana Pavasovic

**Affiliations:** 1 School of Biomedical Sciences, Faculty of Health, Queensland University of Technology, GPO Box 2434, Brisbane, Qld 4001, Australia; 2 School of Earth, Environmental and Biological Sciences, Science and Engineering Faculty, Queensland University of Technology, GPO Box 2434, Brisbane, Qld 4001, Australia; 3 Institute for Molecular Biosciences, University of Queensland, St Lucia, Qld 4072, Australia

**Keywords:** *Nerita melanotragus*, *De novo* assembly, Transcriptome, Heat shock protein, Ion torrent

## Abstract

**Background:**

The sequencing, *de novo* assembly and annotation of transcriptome datasets generated with next generation sequencing (NGS) has enabled biologists to answer genomic questions in non-model species with unprecedented ease. Reliable and accurate *de novo* assembly and annotation of transcriptomes, however, is a critically important step for transcriptome assemblies generated from short read sequences. Typical benchmarks for assembly and annotation reliability have been performed with model species. To address the reliability and accuracy of *de novo* transcriptome assembly in non-model species, we generated an RNAseq dataset for an intertidal gastropod mollusc species, *Nerita melanotragus,* and compared the assembly produced by four different *de novo* transcriptome assemblers; Velvet, Oases, Geneious and Trinity, for a number of quality metrics and redundancy.

**Results:**

Transcriptome sequencing on the Ion Torrent PGM™ produced 1,883,624 raw reads with a mean length of 133 base pairs (bp). Both the Trinity and Oases *de novo* assemblers produced the best assemblies based on all quality metrics including fewer contigs, increased N50 and average contig length and contigs of greater length. Overall the BLAST and annotation success of our assemblies was not high with only 15-19% of contigs assigned a putative function.

**Conclusions:**

We believe that any improvement in annotation success of gastropod species will require more gastropod genome sequences, but in particular an increase in mollusc protein sequences in public databases. Overall, this paper demonstrates that reliable and accurate *de novo* transcriptome assemblies can be generated from short read sequencers with the right assembly algorithms.

## Background

The phylum Mollusca is a highly abundant group of marine animals accounting for over 23% of all marine species [1,2], and as such are a dominant taxa of many marine ecosystems. These organisms are also of significant economic importance as a source of bioactive compounds in addition to being aquaculture and fisheries commodities. Molluscs also serve as valuable models for behavioural neurobiology, respiration and feeding in animals [3-6]. Consequently, molluscs are very important both economically and ecologically. However, genomic resources remain scarce for Mollusc species, with transcriptome data availble for only select species such as *Crassostrea gigas, Macoma balthica, Aplysia californica* and *Lymnaea stagnalis*[7-10] As a result, this group of organisms remains relatively poorly studied at the genomic level.

Research into the genomics of gastropod molluscs has lagged, because genomic resources are not developed for many species. Next generation sequencing platforms such as Illumina and Ion Torrent have recently been used to rapidly characterise transcriptome sequences from a number of non-model organisms [11-14]. In this study, we use the Ion Torrent platform, an efficient and low cost platform to sequence the *Nerita melanotragus* transcriptome, a non-model species without a reference genome.

Precise and accurate *de novo* assembly and annotation of transcriptomes, however, is a commonly overlooked but critically important step for assemblies generated from short reads (~100-150 bp). Recently, many *de novo* assemblers have been developed with specific algorithms for transcriptome assembly from Illumina short reads, nonetheless their effectiveness for *de novo* assembly of Ion Torrent data remains relatively unexplored as the Ion Torrent technology is newer and still gaining acceptance in the research community. Accurate assembly of short reads into longer contigs is important for the functional annotation of ESTs in non-model organisms. In fact, one of the major challenges for genomic research in mollusc species is that many genes remain unannotated. In this study, we address these issues by comparing the performance of a number of short read *de novo* transcriptome assemblers using Ion Torrent sequence data in *Nerita melanotragus*.

The black nerite (*N. melanotragus*) is a marine gastropod within the phylum Mollusca. This species inhabits the intertidal zone and has a large geographic distribution from central Queensland, Australia to southern New Zealand [15]. As a consequence, the environmental conditions that this species is exposed to change temporally and spatially, on both micro and macro geographic scales. Thus, this organism is a good candidate to explore the genetic and gene expression changes, which allow it to persist in such a dynamic environment. Little is known about adaptation genetics and plastic gene expression changes in *N. melanotragus* due an explicit lack of genomic resources. To address this issue, we report a first *de novo* assembly of the *N. melanotragus* transcriptome. Specifically, this study focuses on addressing the following aims: 1. to generate genomic resources for this species through whole organism transcriptome sequencing; and 2. to assess the accuracy and precision of four different short read *de novo* transcriptome assemblers.

## Methods

**Figure 1:**
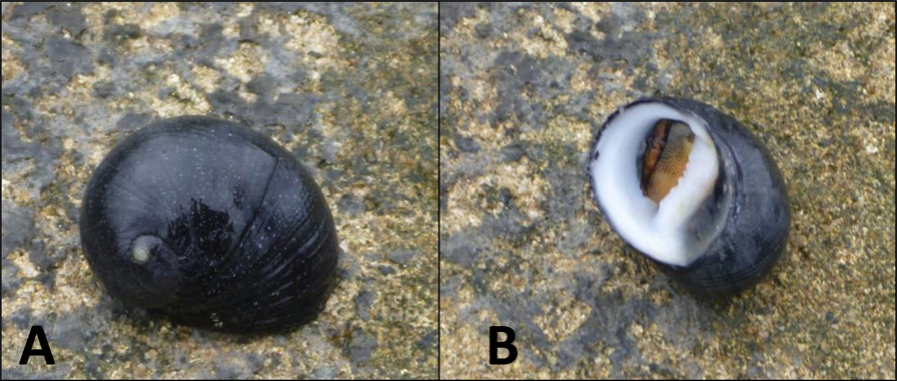
**Black nerite *(N. melanotragus)*.** Black nerite displaying external morphology **(A)** and black nerite displaying tan/brown colouration of its operculum **(B)**.

Black nerite (*N. melanotragus*), (Figure 1A), individuals were collected from the rocky intertidal zone at Caloundra, Queensland, Australia (26°48′17“S 153°8′28”E). Ethics approval and collection permits/licenses were not required for specimen collection. Individual animals were classified as *N. melanotragus* based on operculum colour [16]. A single individual was snap frozen in liquid nitrogen (LN_2_) and stored at -80°C until RNA extraction. The frozen tissue sample from the whole organism was homogenised in LN_2_ and total RNA was extracted using a Trizol/Chloroform extraction protocol followed by a clean up using an RNeasy Minikit (Qiagen). RNA samples were treated using Turbo DNase (Ambion), according to manufacturer’s protocol.

To check the quantity and integrity of the total RNA, the sample was run on a Bioanalyzer 2100 RNA Nano chip (Agilent Technologies). Messenger RNA was isolated from total RNA using the Dynabeads mRNA Purification Kit (Life Technologies). A Bioanalyzer 2100 Pico chip (Agilent Technologies) was used to determine the quality and quantity of isolated mRNA.

High quality mRNA (100–500 ng) was fragmented into 200–700 bp pieces using RNase III (Life Technologies) and Agincourt beads were used to remove small RNA fragments. The yield and size distribution of fragmented RNA was determined on a Bioanalyzer 2100 using a RNA 6000 Pico chip (Agilent Technologies). Library construction was conducted as per the Ion Total RNA-Seq Kit (Life Technologies) for whole transcriptome libraries and cDNA yield and size was determined using a Bioanalyzer 2100 high sensitivity DNA chip.

Template preparation for sequencing was conducted according to the OneTouch Ion™ Template Kit (Life Technologies). Ion Torrent sequencing was conducted using the Ion PGM^TM^ 200 Sequencing Kit (Life Technologies) on an Ion Torrent Personal Genome Machine (PGM^TM^, Life Technologies) using a 318-chip (Ion 318^TM^ chip, Life Technologies).

Raw sequencing reads were converted to FastQ files and assessed for quality scores. Reads were accepted based on a quality threshold (Q > 20, ambiguous bases less than 1%), and adapter sequences were removed prior to downstream analyses. To critically assess the quality of this Ion Torrent data we undertook a number of analytical approaches described by [17] including the sequencing depth and coverage for expressed genes from the publically available mitochondrial genome of *N. melanotragus* using Geneious Pro (Version 5.6) [18].

High quality reads were assembled into contiguous sequences (contigs) using four different short read *de novo* assemblers, which included following: 1) Geneious Pro (Version 5.6) [18]; 2) Velvet, short read assembler, Version 1.2.08 [19]; 3) Oases short read assembler, Version 0.2.08 [20] and 4) Trinity short read assembler [21]. All assemblers except Geneious used the following assembly parameters: kmer hash length = 25, coverage cut-off = 3x; minimum contig length = 100 bp. In the Geneious software, kmer hash length and coverage cut-off could not be changed, so default settings were used with a minimum contig length of 100 bp. The assembly created by the four different assemblers were compared for three different parameters; the number of contigs produced, the N50 statistic and the longest contig to determine which assembler performed best.

To determine the redundancy of the assemblies produced by the four different assemblers we remapped our assembled datasets to the mitochondrial reference gene set from *N. melanotragus* (publically available from NCBI). All contigs produced by the four different assemblers were remapped to this gene set and the overall number of hits was calculated as a quality score.

Following contig generation, the transcriptome assemblies for Trinity and Oases were referenced to the NR database at NCBI as BLASTx queries using the Blast2GO® software suite [22]. In order to be used in downstream analyses, BLASTx hits had to be below an E-value of 1 × 10^-6^. Annotation analyses were performed at levels 2 and 3. The Blast2GO® software suite was also used to predict the functions of contigs with BLASTx hits and assign Gene Ontology (GO) terms to the sequences. To determine which of the short read assemblers produced the best assembly of our Ion Torrent data, we compared the BLAST and annotation success of these different datasets.

To validate the reliability and accuracy of our assembly and annotation, we randomly chose two contigs (annotated as β (beta) - actin and NADH dehydrogenase subunit 5) and designed primers for PCR and Sanger sequencing. Primers were designed using BatchPrimer3 (Version 1.0) using settings as per [23]. Details of the primer sequences are provided in the supplementary material for this paper (Additional file 1: Table S1). PCR was performed according to the MyTaq^TM^ (Bioline) protocol with the following concentrations of reagents 1 × PCR Buffer, 1 μM of each primer, 0.1 units of MyTaq^TM^ DNA Polymerase (Bioline) and 20 ng of template genomic DNA (from same individual that was sequenced) in a total volume of 25 μL. PCR conditions were as follows: 3 min at 94°C, followed by 30 cycles of 30 sec at 94°C, 30 sec at 52°C, 30 sec 72°C, 3 min at 72°C. Amplicons were purified using the Isolate PCR Kit (Bioline) and cycle sequencing was carried out using BigDye® Terminator v3.1 Cycle Sequencing Kit (Life Technologies). After a MgSO_4_ clean-up, the amplicons were run on an ABI 3500 Genetic Analyzer (Life Technologies). Sequences were visualised and edited by eye using Geneious Pro Version 5.6. These sequences were then used as BLASTn queries against the nucleotide database at NCBI and were compared for differences against the original sequences.

## Results

### Ion torrent sequencing and reads assembly

Transcriptome sequencing of mRNA from *N. melanotragus* on the Ion Torrent PGM platform generated a total of 249.67 Mbp of sequence from 1,883,624 raw reads. Mean length of reads was 133 bp, with the longest read being 392 bp. Sequence reads that did not meet our strict quality criteria (Q < 20, ambiguous bases > 1%) were excluded and 84.19 Mb of high quality data was retained for downstream analysis, as low quality bases are likely to reduce the accuracy of transcriptome assemblies.

Based on high quality reads, a total of 112 762, 78 306, 10 886 and 3 090 contigs were generated using the following four different assemblers Geneious, Velvet, Trinity and Oases, respectively (Table 1). Overall the Oases assembly produced the longest contig at 1700 bp closely followed by Trinity at 1618 bp. The longest contigs produced by Geneious and Velvet were over 700 bp shorter than the other two *de novo* assemblers (Table 1). The length of the N50 statistic in the Geneious, Velvet and Oases assemblies were noticeably shorter than that calculated for the Trinity assembly (Table 1). Average contig length showed a similar trend, with the Trinity assembly also having the longest average contig length.

**Table 1:** Assembly quality metrics

**Assembly Statistic**	**Assembler**			
	**Oases**	**Trinity**	**Velvet**	**Geneious**
Number of contigs	3 090	10 886	78 306	112 762
Average contig length	175	293	111	140
Longest contig	1 700	1 618	458	711
N50	149	258	107	124

Assembly statistics for the transcriptomes produced by the four different short read *de novo* assemblers.

Both Velvet and Geneious assemblies had a greater number of contigs remapped to the mitochondrial expressed gene set with 450 and 420 hits respectively, compared to 37 and 25 hits for Trinity and Oases, respectively. The coverage of the contigs produced by both Trinity and Oases was greater than 95%, while the coverage produced by the Geneious and Velvet contigs was less than 55% for both assemblies. The Geneious and Velvet assemblies were found to be highly redundant and produced more fragmented contigs, consequently they were removed from further analyses.

Remapping of high quality reads to the transcribed genes in *N. melanotragus* mitochondrial genome resulted in an assembly with an average of approximately 374 × read depth and greater than 99.5% coverage. The sequencing depth was highest for the 16S rRNA gene with >2000 × read depth. All genes had coverage of greater than 98% with the lowest coverage occurring in NADH dehydrogenase subunit 2, which contained a 40 bp region with no coverage.

### Functional annotation of contigs

Of the 10886 and 3090 contigs queried against the NR database only 2069 and 475 returned significant hits at greater than 1 × 10^-6^ stringency (Table 2). This meant that approximately 19 and 15.4% of contigs could be assigned putative functions for the Trinity and Oases assemblies, respectively.

**Table 2:** Annotation results

**Annotation category**	**Annotation result (number of sequences)**	
	**Trinity**	**Oases**
Without blast result	0 (0%)	0 (0%)
Without blast hits	8823 (81%)	2615 (84.6%)
With blast result	301 (2.7%)	66 (2.1%)
With mapping result	177 (1.6%)	28 (0.9%)
Annotated sequences	1585 (14.5%)	381 (12.3%)
Total sequences	10886	3090

The number of contigs allocated to different annotation categories for the Trinity and Oases assemblies.

**Figure 2:**
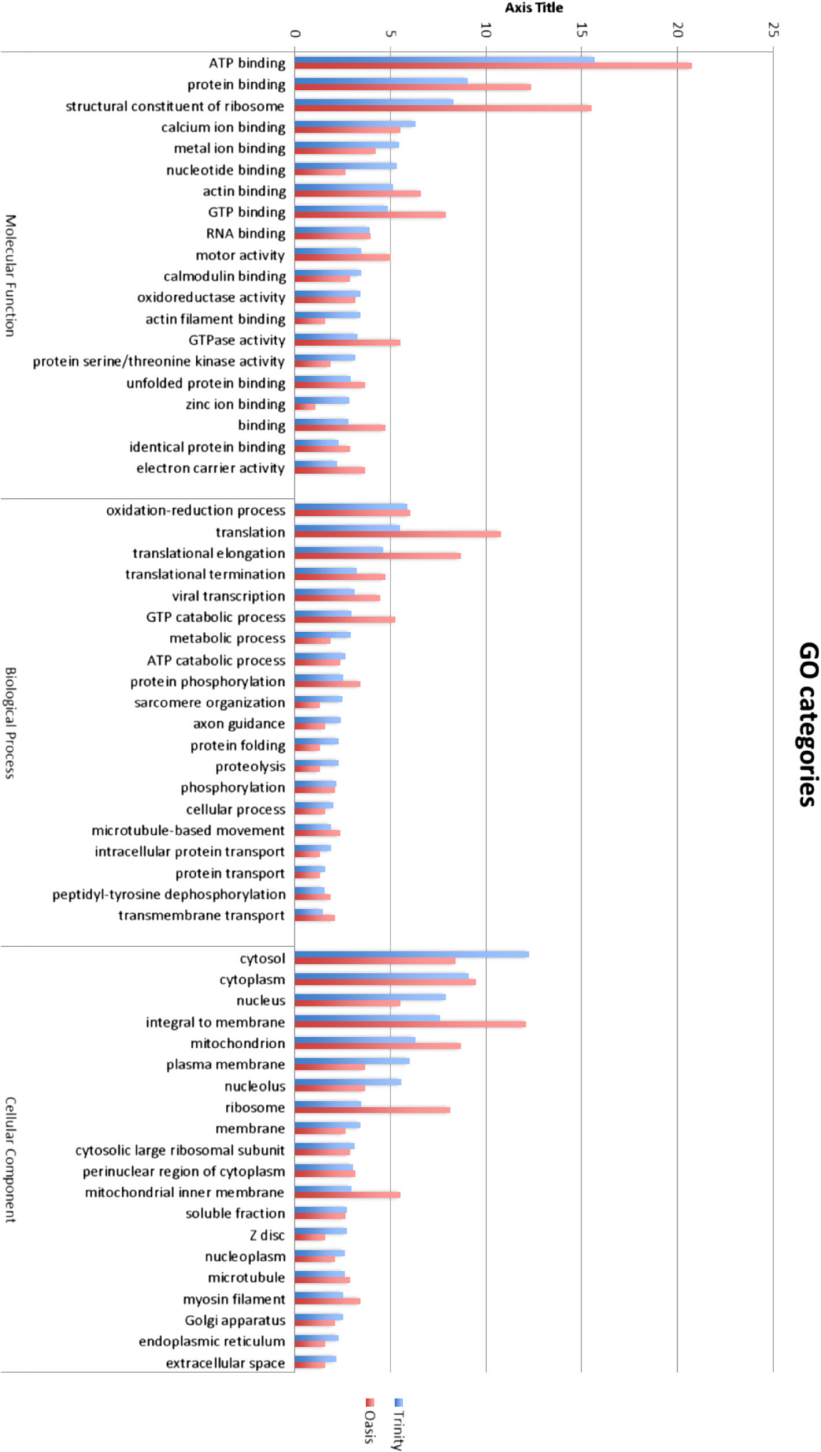
**GO category assignment.** Comparative analysis and functional classification of the top 20 GO terms for the Trinity and Oases assembly.

**Figure 3:**
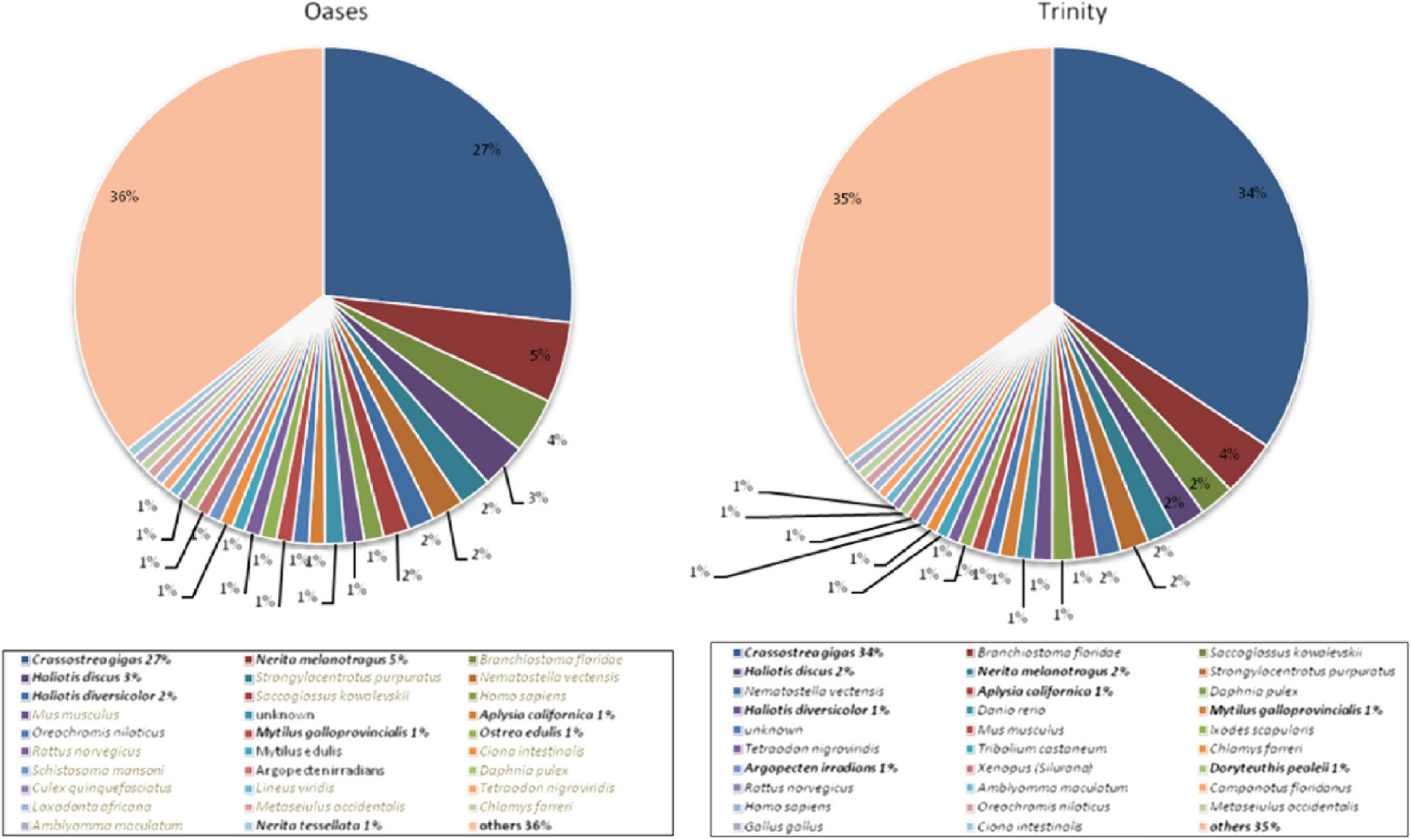
**BLAST top hit species distribution.** The 20 species most commonly represented in BLAST hits for Trinity and Oases assemblies.

Despite the limited number of contigs assigned BLAST hits, the contigs generated by both Trinity and Oases captured a broad range of different types of transcripts, as indicated by the variety of Gene Ontology (GO) terms assigned. A total functional annotation dataset is provided in (Additional file 1: Figure S1 and Figure S2), and here we report only the results for the top 20 GO terms for each category (Figure 2). The GO category with the highest number of terms assigned molecular function, followed by cellular component while biological process had the least contigs assigned terms. The most commonly assigned GO terms in the molecular function GO category were the housekeeping genes involved in ATP binding, protein binding and structural constituent of ribosome for both assemblies (Figure 2). Oxidation-reduction process, translation and translational elongation were the most commonly assigned terms for the biological process GO category. The three most commonly assigned GO terms for cellular component were cytosol, cytoplasm and nucleus, and cytoplasm, integral to membrane and mitochondrion for the Trinity and Oases assembly, respectively. Over 65% of BLAST hits were made up of different mollusc species. The Pacific oyster, *C. gigas*, which made up 27 and 34% of BLAST hits for the Oases and Trinity assemblies, dominated top BLAST hits. Other molluscs including *Haliotis discus* and *H. diversicolor* were also in the top four species that made up top BLAST hits for both assemblies (Figure 3).

### PCR validation of contigs

The PCR primer pairs designed for beta-actin and NADH dehydrogenase subunit 5 amplified a single product of the correct size, images provided in the (Additional file 1: Figure S1). High quality sequence was obtained for both amplicons using both forward and reverse primers. The BAC and NAD sequences were assigned top nucleotide blast hits for beta-actin from *Aplysia californica* and NADH dehydrogenase subunit 5 from *N. melanotragus*, respectively. Protein blast confirmed this result with an E-value of greater than 1 × 10^-27^. Alignment of the beta-actin and NADH dehydrogenase subunit 5 sequences to the contigs from which they were designed resulted in a perfect match for beta-actin and the presence of a single one base pair indel for NADH dehydrogenase subunit 5, in an adenosine homopolymer region.

## Discussion

The availability and throughput of next generation sequencing technologies has enabled the rapid and efficient sequencing of transcriptomes for model and non-model species. The majority of *de novo* transcriptome assemblies in non-model organisms have in the past been produced using the long reads (300-600 bp) generated using Roche 454 [24]. With the recent developments in sequencing technology, short read sequencers (90-400 bp), such as Illumina and Ion Torrent, are starting to be more commonly used for the generation of large next generation sequencing data sets, as the costs are much lower for the same output [25]. Consequently, the use of short read sequencers to generate *de novo* transcriptome assemblies for non-model organisms may lead to a more complete gene set for these species at a lower cost. The reliability of *de novo* transcriptome assemblies generated from short read sequencers, however, needs to be validated to ensure that assemblies are accurate and won’t compromise the downstream applications of next generation sequencing projects. In this paper we compare a number of *de novo* assemblers to demonstrate that short read RNA-seq data generated by an Ion Torrent PGM^TM^ sequencing system can reliably and accurately be assembled for a non-model organism.

Accurate *de novo* assembly of transcriptomes is crucial for next generation sequencing projects in non-model organisms. Of particular importance is finding short read assembly algorithms that produce accurate and reliable assemblies from the short reads produced by Ion Torrent or Illumina sequencers. In our comparison of four different short read assemblers using Ion Torrent data, we found that Trinity and Oases outperformed Velvet and Geneious in all performance metrics, including longer N50 and average contig lengths, producing fewer and longer contigs and having less redundant contigs. Overall, these results are similar to those obtained when comparing Trinity or Oases against other short read assemblers in simulation studies and empirically, with Illumina data [26]. Even though Trinity and Oases outperformed the other assemblers in all metrics, their respective assemblies performed better for different quality metrics. For example, Trinity had a longer N50, while Oases produced fewer contigs with less redundancy.

The *de novo* assemblies generated by both Trinity and Oases produced N50 and average contig lengths similar to many past transcriptome sequencing studies [11-13]. The N50 and average contig size of our Trinity assembly are also similar to that reported for the recently sequenced transcriptome of the common pond snail, *Radix balthica*[27]. In contrast, the N50 and average contig size (>1200 bp) reported for a transcriptome sequence of a different pond snail, *Lymnaea stagnalis*[4] are 6x larger than that of the Trinity assembly for our dataset. A few differences between our transcriptome assembly and that for the *L. stagnalis* transcriptome assembly may account for this difference. Firstly, their dataset had approximately 40x more 100 bp Illumina sequences than in our study. Secondly, the *L. stagnalis* study was conducted for a single tissue type, the central nervous system, while our study utilized the whole animal. These two factors may explain much of the difference in N50 and average contig length between the two studies. Therefore, it is highly likely that *de novo* assemblies generated with a similar amount of Ion Torrent data could result in assemblies with more comparable N50 and average contig lengths.

The blast and annotation success for both the Trinity and Oases assemblies was quite low (15-19%). This level of annotation success is much lower than that often reported in the literature even for non-model species [25]. The level of annotation success achieved in this study, however, is in a similar range to that reported for two recently sequenced gastropod transcriptomes using short read technologies (*R. balthica* 17% and *L. stagnalis* 20.1%) [27,4]. One of the reasons put forward to explain the low degree of annotation success is the fact that few reference genome sequences exist for mollusc species [4]. We also hypothesise that an improvement in annotation success of gastropod species will require more representative gastropod reference genome sequences and an increase in mollusc protein sequences in public databases.

In this paper we describe an EST collection generated by Ion Torrent sequencing and *de novo* assembly to characterize the transcriptome of a non-model gastropod species, *N. melanotragus*. This marine gastropod is a common component of the intertidal zone on rocky substrates and distributed from Mackay (Queensland) to southern Tasmania and New Zealand. Across this large geographic distribution *N. melanotragus* spans a number of environmental gradients such as clines in water temperature, oxygen concentration and substrate type [16]. The environment of this species also varies temporally on a micro geographic scale between tidal cycles and consequently *N. melanotragus* is exposed to large changes in a number of environmental factors including temperature, pH, salinity and dissolved oxygen [16]. Therefore generating genomic datasets such as in this study is crucial as we focus our research efforts towards understanding genetic and gene expression changes that have allowed this intertidal species to adapt and cope with dramatic fluctuations in environmental conditions.

## Conclusion

The large number of contigs that we have annotated and functionally characterized in this study provides a first step towards a systems biology approach to physiological genomics in gastropod species. By identifying a wide variety of genes from a number of different GO classes we can now determine which genes are important for adaptation across broad environmental changes and for stress response to micro geographic environmental fluctuations. This is very important because we still know remarkably little about the physiology and evolution of many marine organisms and in particular the physiological basis of adaptation to both spatial and temporal environmental variation in intertidal zone species [28,29].

### Availability of supporting data

The raw sequence reads supporting the results of this article are available in the short reads archive under the accession number SRR1054996 (http://www.ncbi.nlm.nih.gov/sra/?term=SRR1054996). PCR validation gene sequences are available under the accession number KM025036 (beta-actin) and KM025037 (NADH dehydrogenase subunit 5).

## Competing interests

The authors declare that they have no competing interests.

## Authors’ contributions

SA performed experimental work and sequencing analysis. PP, EG and AP designed the experiment, helped with data interpretation and provided guidance throughout the whole study. All authors have read and approve of the manuscript.

## Supplementary Material

Additional file 1 **Table S1.** Primer sequences for β-actin and NADH dehydrogenase subunit 5. Primers were designed to validate the reliability and accuracy of our assembly and annotation. **Figure S1** Visualisation of PCR products. Agarose electrophoresis gel showing two candidate genes beta-actin (A) and NADH dehydrogenase (B) (Molecular marker Hyperladder IV). **Figure S2***Nerita melanotragus* transcriptome functional annotation based on Trinity Blast2GO analysis. Functional annotation results indicate the relative amount of each category of contigs with protein hits. The results are summarized as follows: Biological Process (BP), Molecular Function (MF) and Cellular Component (CC). **Figure S3***Nerita melanotragus* transcriptome functional annotation based on Oases Blast2GO analysis. Functional annotation results indicate the relative amount of each category of contigs with protein hits. The results are summarized as follows: Biological Process (BP), Molecular Function (MF) and Cellular Component (CC). Click here for file
