# Supplementary material for: Assembly and annotation of a non-model gastropod (Nerita melanotragus) transcriptome: a comparison of De novo assemblers
Source: BMC Res Notes. 2014 Aug 1;7:488. doi: 10.1186/1756-0500-7-488 (PMC4124492; doi:10.1186/1756-0500-7-488)

**Additional file 1**

TABLE S1. **Primer sequences for β-actin and NADH dehydrogenase subunit 5**. Primers were designed to validate the reliability and accuracy of our assembly and annotation.

| **Primer name** | **Primer sequence** | **Fragment length (bp)** |
| --- | --- | --- |
| Beta-actin-Nm (F) | GAAGCTGTGCTATGTTGTCCTC | 450 |
| Beta-actin-Nm (R) | GATCTTGATCTTCATGGTGCTG | 450 |
| NADH (F) | GGCGCATTAGCATCTCAAAT | 414 |
| NADH (R) | GCTCCTGCAAGGGTAACTGA | 414 |

Figure S1. **Visualisation of PCR products.** Agarose electrophoresis gel showing two candidate genes beta-actin (A) and NADH dehydrogenase (B) (Molecular marker Hyperladder IV)


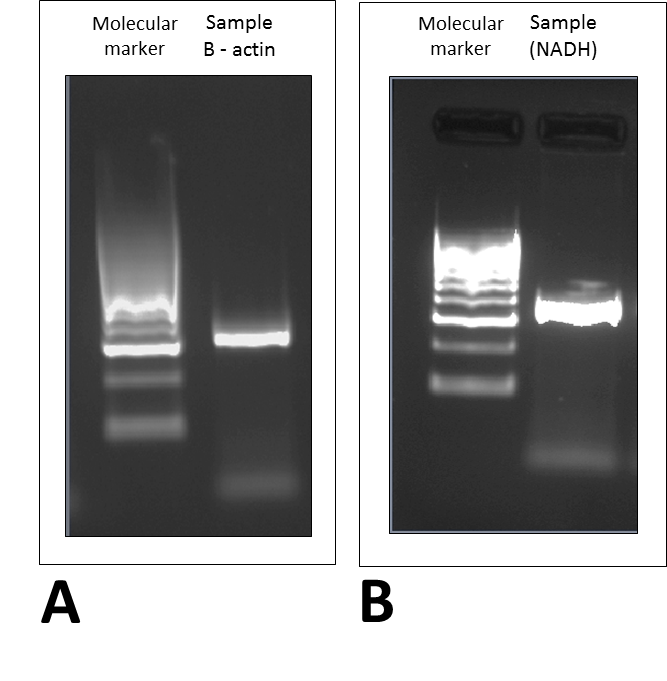


500 bp

300 bp

200 bp

100 bp

Figure S2. ***Nerita melanotragus* transcriptome functional annotation based on Trinity Blast2GO analysis.** Functional annotation results indicate the relative amount of each category of contigs with protein hits. The results are summarized as follows**:** Biological Process (BP), Molecular Function (MF) and Cellular Component (CC).


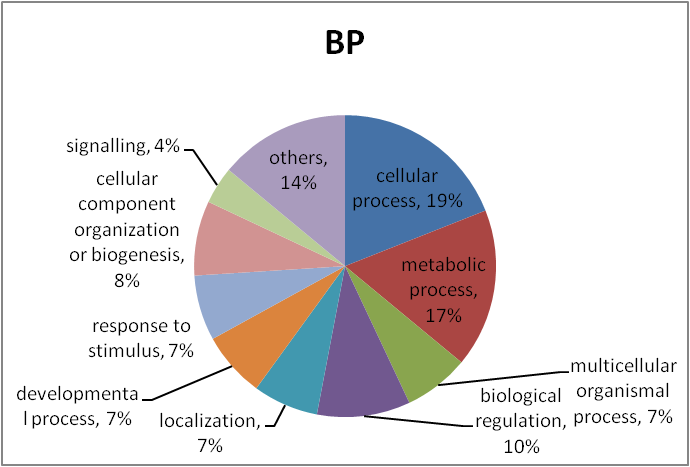


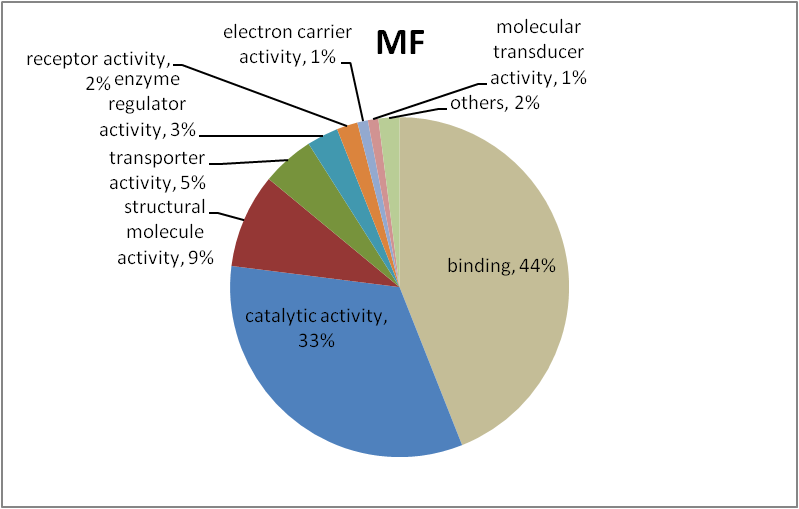

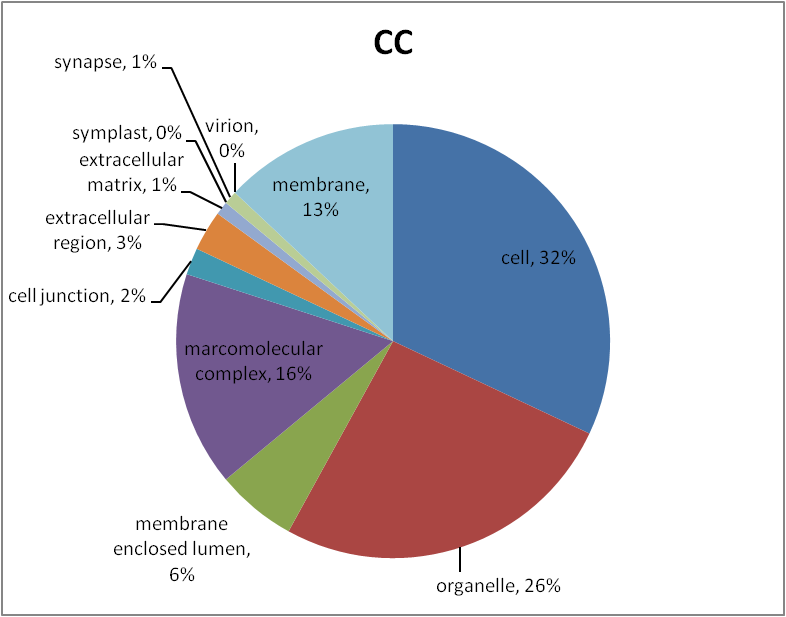


Figure S3. ***Nerita melanotragus* transcriptome functional annotation based on Oases Blast2GO analysis.** Functional annotation results indicate the relative amount of each category of contigs with protein hits. The results are summarized as follows**:** Biological Process (BP), Molecular Function (MF) and Cellular Component (CC).


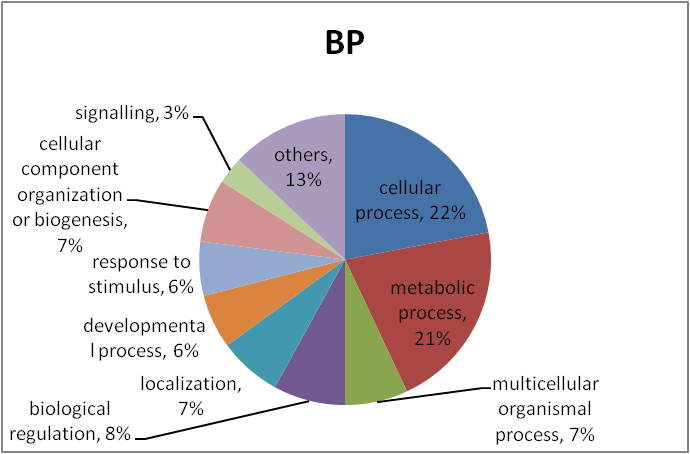


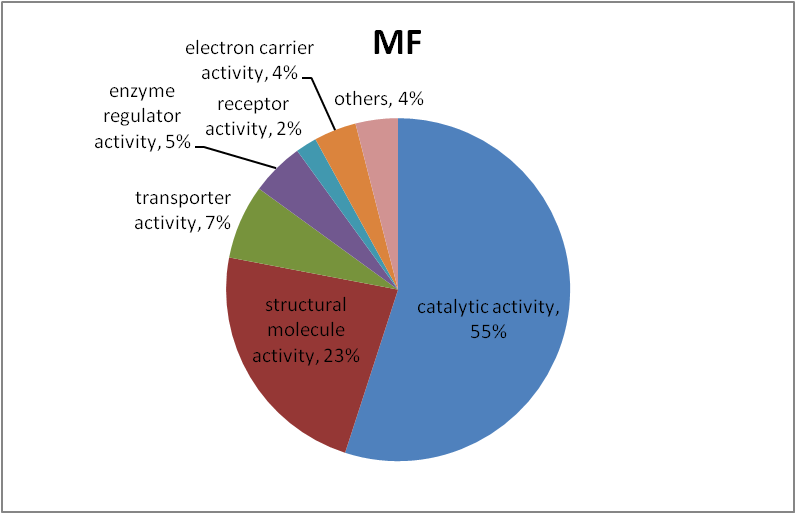

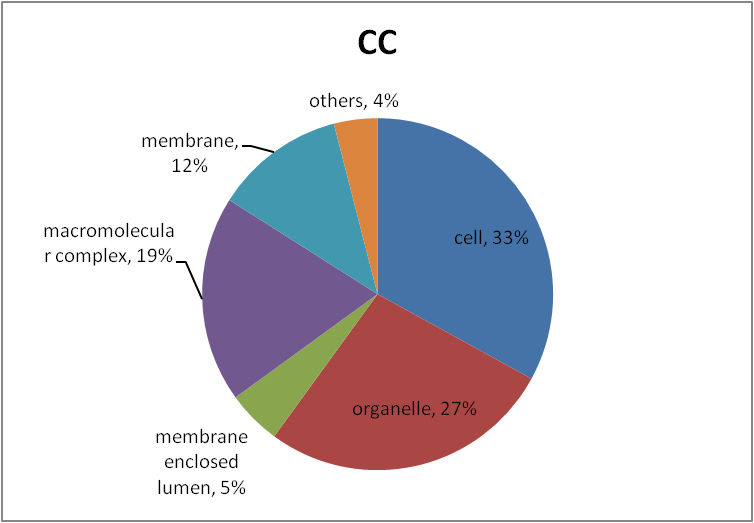

Supplement: Additional file 1 — Table S1. Primer sequences for β-actin and NADH dehydrogenase subunit 5. Primers were designed to validate the reliability and accuracy of our assembly and annotation. Figure S1 Visualisation of PCR products. Agarose electrophoresis gel showing two candidate genes beta-actin (A) and NADH dehydrogenase (B) (Molecular marker Hyperladder IV). Figure S2Nerita melanotragus transcriptome functional annotation based on Trinity Blast2GO analysis. Functional annotation results indicate the relative amount of each category of contigs with protein hits. The results are summarized as follows: Biological Process (BP), Molecular Function (MF) and Cellular Component (CC). Figure S3Nerita melanotragus transcriptome functional annotation based on Oases Blast2GO analysis. Functional annotation results indicate the relative amount of each category of contigs with protein hits. The results are summarized as follows: Biological Process (BP), Molecular Function (MF) and Cellular Component (CC). [file 1756-0500-7-488-S1.docx]
